# Phenotypic Switching of *Staphylococcus aureus* Mu50 Into a Large Colony Variant Enhances Heritable Resistance Against β-Lactam Antibiotics

**DOI:** 10.3389/fmicb.2021.709841

**Published:** 2021-10-07

**Authors:** Yajun Sun, Miaomiao Liu, Mingze Niu, Xin Zhao

**Affiliations:** ^1^College of Animal Science and Technology, Northwest A&F University, Yangling, China; ^2^Department of Animal Science, McGill University, Montreal, QC, Canada

**Keywords:** *Staphylococcus aureus*, MRSA, phenotype switching, large colony, β-Lactams, *lcpA*, *spa*

## Abstract

Phenotypic heterogeneity within a bacterial population may confer new functionality and allow microorganisms to adapt to fluctuating environments. Previous work has suggested that *Staphylococcus aureus* could form small colony variants to avoid elimination by therapeutic antibiotics and host immunity systems. Here we show that a reversible non-pigment large colony morphology (Mu50∆*lcpA*-LC) was observed in *S. aureus* Mu50 after knocking out *lcpA,* coding for the LytR-CpsA-Psr family A protein. Mu50∆*lcpA*-LC increased resistance to β-lactam antibiotics, in addition, the enlarged cell size, enhanced spreading ability on solid medium, and reduced biofilm formation, suggesting better abilities for bacterial expansion. Moreover, the expression of *spa* encoding protein A was significantly increased in Mu50∆*lcpA*-LC. This study shows that besides the small colony variants, *S. aureus* could fight against antibiotics and host immunity through phenotype switching into a large colony variant.

## Introduction

*Staphylococcus aureus* is an important human opportunistic pathogen that commonly colonizes the skin, mucosal surfaces, and soft tissues. When host barriers are disrupted, *S. aureus* can cause diverse clinical syndromes, ranging from skin abscess to more serious infections such as pneumonia, bacteremia, endocarditis, and osteomyelitis (Turner et al., 2019). *S. aureus* has been notoriously able to acquire resistance to a variety of antibiotics. The targets for β-lactam antibiotics are known as penicillin-binding proteins (PBPs). β-Lactam antibiotics inhibit the last step in peptidoglycan synthesis by acylating the penicillin-binding proteins involved in cross-linking peptides to form peptidoglycan, which is a vital constituent of the bacterial cell wall. However, the increasing use of β-lactams has resulted in the worldwide spread of the methicillin-resistant *S. aureus* (MRSA; Lee et al., 2018). The resistance of MRSA is usually conferred by acquisition of a low-affinity penicillin-binding protein 2A (Hartman and Tomasz, 1984).

Other proteins such as LytR-CpsA-Psr (LCP) family members than PBPs also play important roles in bacterial cell wall synthesis. *S. aureus* possesses three *lcp* genes, including *lcpA* (also named *msrR*), *lcpB*, and *lcpC*. These three LCP proteins of *S. aureus*, in particular LcpA, could catalyze the linkage of wall teichoic acid (WTA) to synthetic uncrosslinked peptidoglycan (PGN) oligomers *in vitro* (Schaefer et al., 2017; Schaefer et al., 2018). LcpA can glycosylate the wall attachment protein GspA in the gram-positive bacterial *Actinomyces oris* (Siegel et al., 2019). However, this latter function has not been demonstrated in *S. aureus*. Similarly, LcpC in *S. aureus* catalyzed attachment of the capsular polysaccharide to PGN (Rausch et al., 2019). In addition, *lcpA* contributed to oxacillin resistance in two MRSA strains COLn and USA300 (Hubscher et al., 2009; Schaefer et al., 2017) and a methicillin-susceptible *S. aureus* (MSSA) MSSA1112 (Hubscher et al., 2009). The deficiency in *lcpC* reduced the β-lactam resistance in two MRSA strains BA01611 and Mu50 and an MSSA strain Newman (Li et al., 2020). Interestingly, BA01611∆*lcpC* had decreased teicoplanin resistance but Mu50∆*lcpC* had increased teicoplanin resistance (Li et al., 2020). Teicoplanin is a semisynthetic glycopeptide antibiotic with a spectrum of activity similar to vancomycin. Both teicoplanin and vancomycin inhibit cell wall biosynthesis. Mu50 is a vancomycin-intermediate *S. aureus* strain (VISA), which possesses active cell wall biosynthesis, leading to a thicker cell wall than the vancomycin-susceptible *S. aureus* (VSSA) strains (Cui et al., 2000; Katayama et al., 2016). Indeed, BA01611, a VSSA strain, has a thinner envelope than that of Mu50 (Li et al., 2020). It seems that the deficiency of *lcpC* had different effects on Mu50 and BA01611. Thus, we hypothesized that *lcpA* might also have different effects on resistances in Mu50 and BA01611. To confirm the hypothesis, we constructed the Mu50∆*lcpA* and BA01611∆*lcpA* strains. We found that the *lcpA* deletion had different effects on resistances between these two strains. In addition, we observed co-existence of larger colonies and normal colonies in Mu50∆*lcpA* strain populations, in comparison with the wild type (WT) of Mu50. This type of phenotype variation has never been reported in *S. aureus* before.

Most microorganisms such as *S. aureus* live in groups of varying degrees of complexity. Various subpopulations within the group are responsible for the division of labor, which provide bacteria populations more benefits to survive different host environments and therapeutic antimicrobial stresses (Ackermann, 2015). Colony morphology variety is one of the most common phenotypic diversity. The subpopulation with distinct colony morphology usually has different phenotypes for antibiotic resistance, motility and virulence. For example, *Clostridioides difficile* could switch their colony morphology between the rough colony and smooth colony. The rough colony variants could swim rapidly and induced more serious infections *in vivo*, while the smooth strain tended to colonize *in vivo* but had attenuated virulence (Garrett et al., 2019). *Streptococcus pneumoniae* underwent spontaneous phase variation between opaque and transparent colony. The transparent variant had more advantages on adhesion to epithelial cells and nasopharyngeal colonization than the opaque counterparts (Li et al., 2016). In *Mycobacterium tuberculosis*, the reversible genetic frameshift mutations could lead to reversible drug resistances and a phenotype switching between large colony and small colony (Safi et al., 2019). In *S. aureus*, the phenotypic switching between normal colony and small colony within an isogenic population commonly occurs. For example, Cui et al. (2012) found the small colony variants (SCVs) Mu50Ω, which had the same general features of the Mu50 genome, could revert back to normal-size colonies, caused by almost half of the chromosome inversion between homologous regions of SaPlm4 and SaPlm1. Guerillot et al. (2019) found that the *S. aureus* NRS384 produced reversible SCVs caused by chromosome reversion between two homologous regions of *hsdM*-*hsdS* genes. Switching into the SCV is a direct response of *S. aureus* to environmental stresses, such as antibiotic stresses and host immune responses. However, whether *S. aureus* uses other types of phenotypic diversity to adapt to environmental changes has not been reported.

Here, we report an unexpected observation of phenotypic variation into large colonies in Mu50∆*lcpA* but not in BA01611∆*lcpA*. This phenotypic variation has not been reported before in other *lcpA* null mutant strains of previous studies (Hubscher et al., 2009; Schaefer et al., 2017). Thus, the objective of this study was to investigate the biological consequence of this variant.

## Materials and Methods

### Bacterial Strains, Plasmids, and Growth Conditions

Bacterial strains and plasmids used in this study are listed in Supplementary Table S1. We used two strains: the representative clinical MRSA-VISA strain Mu50 and the MRSA-VSSA strain BA01611 that isolated from bovine mastitis. *S. aureus* strains were grown in tryptic soy broth (TSB) medium, unless stated otherwise. *E. coli* DH5α cells were cultivated in lysogeny broth (LB) medium with appropriate carbenicillin (Sigma-Aldrich Co., St. Louis, MO, United States).

### Construction of *lcpA* Deletion and Complementary Strains

Deletion and complementation of the *lcpA* gene were performed by the allelic replacement method according to the published method (Wu et al., 2018). Briefly, to establish the *lcpA* null mutants of Mu50 and BA01611, the DNA fragments flanking the *lcpA* were amplified, then ligated into the pKZ2 vector *via* Gibson Assembly, producing p∆*lcpA*. The primer sets were P1/P2 and P3/P4 for plasmid p∆*lcpA* (Supplementary Table S2). The plasmid p∆*lcpA* was electroporated into *S. aureus*. The integration and loss of p∆*lcpA* and the selection of transconjugants were performed as previously described (Wu et al., 2018). The deletion of *lcpA* was verified by PCR amplification (Primers P5 and P6, see Supplementary Table S2) and sequencing.

To construct the complementary strains Mu50∆*lcpA*-LC∷*lcpA*, Mu50∆*lcpA*-NC∷*lcpA* and BA01611∆*lcpA*∷*lcpA*, the *lcpA* was integrated back into the chromosomes of *lcpA* null mutants through the same procedure (Wu et al., 2018). The primer sets were P7/P8 and P10/P11 for p∆*lcpA*-*lcpA*^E146K^ as well as P8/P9 and P10/P11 for p∆*lcpA*-*lcpA* (Supplementary Table S2). Moreover, a restriction site *Xho*1 was inserted behind the *lcpA* transcriptional terminator for distinguishing the complements from the WT strains. The complementation of *lcpA* was confirmed by PCR (Primers P5 and P6, see Supplementary Table S2) and sequencing.

### Assessment of the Phenotype Switching

About 0.5×10^3^CFU of Mu50∆*lcpA* was spread on TSA plates and was grown at 37°C for 48h. Independent colonies of the Mu50∆*lcpA*-LC and Mu50∆*lcpA*-NC were picked from the Mu50∆*lcpA* plate according to colony size and inoculated in TSB for 12h, and then the cultures were diluted and plated on TSA plates. These plates were incubated at 37°C for 48h. This passage was repeated four times. The number of the large colonies and small colonies on the plates of every experiment were used to estimate the switching frequency. The possibility of the phenotypic switching being attributed to the impurity of inoculating bacteria was excluded by the clonal passage.

### Measurement of Staphyloxanthin Production

Each strain was cultured in TSB at 37°C for 24h. Cells were harvested from 1.7ml cultures by centrifugation at 10,000×g and washed twice with PBS. The cell pellets were suspended in 0.4ml methanol and incubated at 55°C for 3min. Cells were removed by centrifugation at 15,000×g. The absorption spectra of the supernatant extracts were measured at OD_465_. The experiment was repeated three times.

### Measurement of Colony Diameter

About 0.5×10^3^CFU per Mu50 isogenic strain was spread on TSA plates and was incubated at 37°C for 48h. To detect the contribution of growth to the colony expansion without passive swelling, these strains were spread on agar plates as described above and were initially incubated at 37°C for 24h and then were incubated subsequently at 4°C for 24h. All plates were imaged at 24h, 36h, and 48h with a Bio-Rad ChemiDoc™ XRS^+^ system (CA, United States) in transmission model. The diameters of 50 random colonies per strain were measured at 24h, 36h, and 48h.

### Colony-Spreading Assay

The bacterial spreading ability was detected as the published method (Kaito and Sekimizu, 2007). Mu50 isogenic strains were incubated in TSB for 12h. TSB supplemented with 0.24% agar was autoclaved and poured onto a plate (90mm diameter). The plates were spotted with the bacterial cultures (2μl) and incubated at 37°C for 48h. The diameters of colonies were measured at 24h, and the colonies were imaged at 24h and 48h using a Bio-Rad ChemiDoc™ XRS^+^ system (CA, United States) in transmission model. The experiment was repeated three times.

### Biofilm Formation Assay

Biofilm formation was performed as the published study (Garcia-Betancur et al., 2017). Briefly, overnight bacterial cultures were diluted (1:200) in TSB plus 0.25% glucose before being added to a 96-well microtiter plate (100μl per well). The plates were incubated at 37°C for 24h without shaking and were then washed three times with PBS, dried at 65°C for 45min, and stained with 0.1% crystal violet for 15min. The stained crystal violet was solubilized for quantitative analysis using acetic acid at 33%. The absorption spectra of suspension were measured at OD_595_. The experiment was repeated three times.

### Antimicrobial Susceptibility Testing

The minimum inhibitory concentration (MIC) of three β-lactams (penicillin, cefazolin, and oxacillin; Tokyo Chemical Industry Co., Shanghai, China) against *S. aureus* were determined firstly by broth microdilution according to the published method (Wiegand et al., 2008). Briefly, three antimicrobial stock solutions were serially diluted twofold in 96-well microtiter plates with fresh Mueller–Hinton (MH) broth containing 2% NaCl. Then bacteria were added into each well, resulting in a final inoculum of 1× 10^6^CFU/ml. Plates were incubated at 37°C for 24h. The MIC was read as the lowest concentration that prevented visible growth. The experiment was repeated three times.

The susceptibilities of three β-lactams and four other antibiotics were further detected using the agar dilution technique following the published method (Wiegand et al., 2008). The bacterial suspensions were serially diluted tenfold in MH broth, giving final bacterial densities ranging from 10^8^CFU/ml to 10^4^CFU/ml. During the preliminary study, 1μl of diluted bacterial suspensions was spotted onto the MH agar plates that had incorporated different concentrations of the antibiotics and 2% NaCl. Plates were incubated at 37°C for 24h before checking the visible colonies. The bacteria showed different degrees of resistance to antibiotics. Then, the antibiotic concentration with the most obvious resistance difference was chosen to repeat this test three times. Images were captured using a Bio-Rad ChemiDoc^™^ XRS^+^ system (CA, United States).

The growth curves of *S. aureus* in TSB with β-lactam antibiotics were performed as the published method (Dilworth et al., 2014) with some modifications. In the preliminary study, overnight bacterial cultures were diluted to a starting density of 10^7^CFU/ml in TSB. Then several different β-lactam concentrations below the minimum inhibitory concentrations (MICs) were added into cultures. Then the antibiotic concentrations at which the mutant strains displayed the most obvious resistant differences were chosen to repeat the experiment. Cultures were shaken at 37°C for 8h and the OD_600_ was determined at 1h intervals. Moreover, the 48-h growth curves in TSB without β-lactams were determined through the same procedure, and the CFUs were determined on antibiotic-free TSA plates. The experiment was repeated three times.

### Transmission Electron Microscopy

Mu50 isogenic strains were grown to stationary phase and were collected by centrifugation. The pellet was incubated in a fixative solution [2.5% glutaraldehyde in 0.05M sodium phosphate buffer (pH 7.4)] overnight at 4°C. Transmission electronic microscopy samples were prepared at the Department of Electron Microscope of XI’AN JiaoTong University Health Science Center. Images were captured on the Hitachi H-7650 instrument. The fraction of the cells with the complete septum was counted according to the septum growth (400 cells per strain were counted). The cell area of 300 cells per strain was measure using the ImageJ plugin.

### RNA Extraction and qRT-PCR Analysis

Bacteria at the exponential phase and stationary phase were harvested and mechanically lysed using glass beads. RNA was isolated from the supernatant using E.Z.N.A.^®^HP Total RNA Kit (Omega, Norcross, United States) and treated with DNaseI (Transgen, Beijing, China). RNA was reverse-transcribed to cDNA using TransScript One-step gDNA Removal and cDNA Synthesis SuperMix (Transgen, Beijing, China). The qRT-PCR was performed using TransStart Tip Green qPCR SuperMix (Transgen, Beijing, China) on a CFX96 Real-Time PCR System (Bio-Rad). The qRT-PCR primers were shown in Supplementary Table S2.

### Statistical Analysis

Statistical analyses were conducted using SPSS 23.0 (SPSS Inc., Chicago, IL). One-way ANOVA followed by LSD or Dunnett’s T3 multiple comparisons test was performed. Significant differences were defined by values of *p* (two tailed)<0.05 (^*^), < 0.01 (^**^), and<0.001(^***^).

## Results

### Reversible Large Colonies With Altered Phenotypes Are Detected in Mu50∆*lcpA* Strain

**Figure 1:**
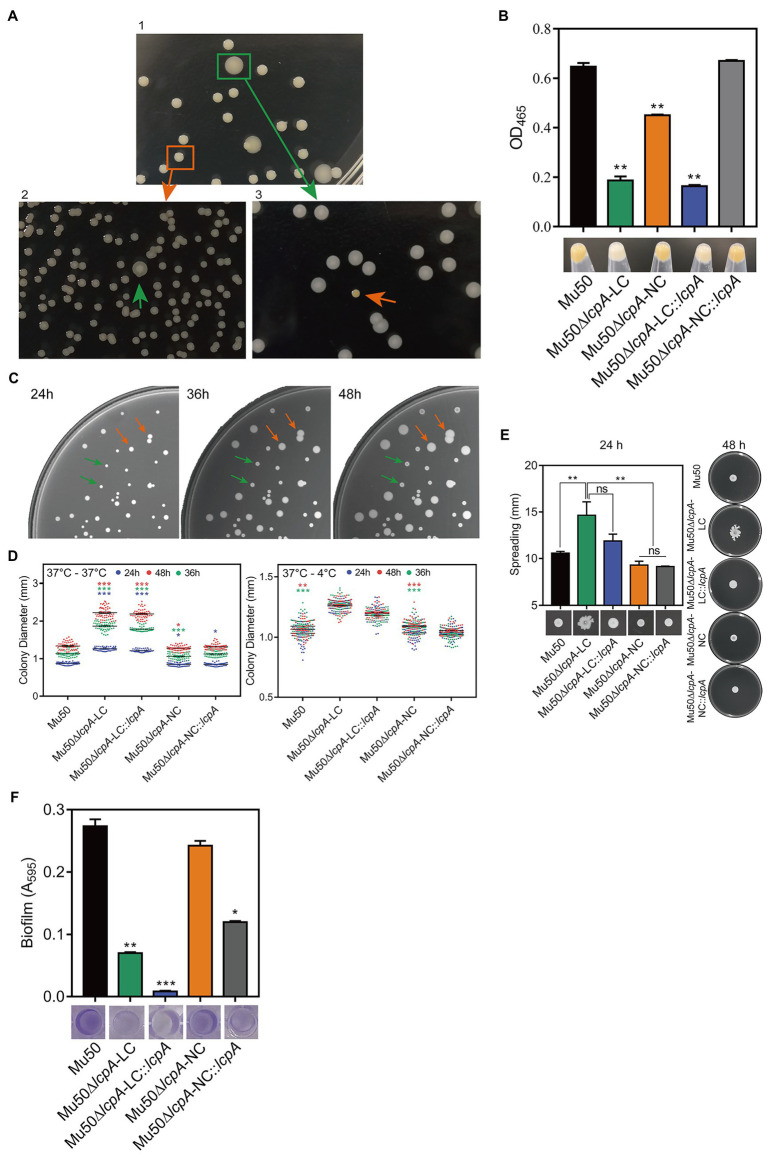
ContinuedFIGURE 1Mu50∆*lcpA* with mixtures of large and normal colonies and the phenotypic characteristics of Mu50 isogenic strains. **(A)** Formation of reversible large and normal colonies by *S. aureus* Mu50∆*lcpA*. Bacteria were grown on TSA plates for 48h to allow differentiation of colony morphology. Populations of Mu50∆*lcpA* consist of two forms of subpopulations that produce large and normal colonies (panel 1). The large and normal colonies can be generated from either Mu50∆*lcpA*-LC or Mu50∆*lcpA*-NC (panel 2 and 3). **(B)** Pigment production of Mu50 isogenic strains. The bottom representative images show the color of cell pellets from overnight grown cultures. **(C)** Heterogeneity in the sizes of Mu50∆*lcpA* colonies. Mu50∆*lcpA* was spread on TSA plates and images were taken after incubation at 37°C for 24h, 36h, and 48h. **(D)** Diameters of Mu50 isogenic strains grown on TSA plates. Left, colonies were incubated at 37°C for 48h. The colony diameter of Mu50 WT was used as a control. Right, colonies were initially grown at 37°C for 24h and subsequently held at 4°C for 24h. The colony diameter of Mu50 WT was used as a control. In all cases, the diameters were measured after 24h, 36h, and 48h. *n*=50. **(E)** Spreading ability of Mu50 isogenic strains. Overnight cultures of Mu50 isogenic strains were dropped on the 0.24% agar plate and incubated 48h at 37°C. The left panel shows the spreading ability at 24h. The right panel shows the spreading ability at 48h. **(F)** Biofilm formation of Mu50 isogenic strains. Biofilm quantitative analysis by crystal violet uptake and the plate was imaged before the absorbance measurement. The green arrow indicates the Mu50∆*lcpA*-LC colonies, the orange arrow indicates the Mu50∆*lcpA*-NC colonies. Error bars correspond to SEM, three independent experiments. One-way ANOVA with LSD or Dunnett’s T3 test for multiple comparisons, ns, no significant, ^*^p<0.05, ^**^p<0.01, ^***^p<0.001.

To investigate the effect of *lcpA* on antibiotic resistance in *S. aureus,* the *lcpA* gene was removed from chromosomes of Mu50 and BA01611 strains, producing the Mu50∆*lcpA* and BA01611∆*lcpA* strains, respectively. During the experiment, we unexpectedly observed that the Mu50∆*lcpA* exhibited two distinct colony morphologies on agar plates: a large flat colony (Mu50∆*lcpA*-LC) and a normal colony (Mu50∆*lcpA*-NC) that was similar to Mu50 WT (Figure 1A, panel 1). Mu50∆*lcpA*-NC had decreased pigment production compared with Mu50 WT, while the pigment of Mu50∆*lcpA-*LC was significantly less than that of Mu50∆*lcpA*-NC (Figure 1B). To test the stability of their colony morphology, a large colony and a normal colony were picked from the Mu50∆*lcpA* agar plate and incubated in tryptic soy broth (TSB) for 12h. Then, each culture was diluted and spread on TSB agar (TSA) plates. Results showed that a few large colonies always appeared in Mu50∆*lcpA*-NC populations, while a few normal colonies always appeared in Mu50∆*lcpA-*LC populations (Figure 1A, panel 2 and 3). From four repeated experiments, the colony count showed that both the large and normal switching colonies appeared in bacteria populations at a frequency of about 0.1%. This heterogeneity in colony morphology was not observed in Mu50 WT and all BA01611 isogenic strains. These results suggest that the deficiency of *lcpA* might induce an unstable phenotype in Mu50.

To further analyze the colony morphology of Mu50∆*lcpA*-LC, the Mu50∆*lcpA* was spread on TSA plates and incubated firstly at 37°C for 24h, then subsequently incubated at 37°C or 4°C for 24h. As shown in Figures 1C,D, bacterial colonies could keep on expanding within 48h at 37°C, and the colony diameter of Mu50∆*lcpA*-LC was significantly larger than Mu50 WT and Mu50∆*lcpA*-NC strains on average. When incubated for 48h at 37°C, the colony diameter of Mu50∆*lcpA*-LC was 66.4% larger than Mu50 WT, while Mu50∆*lcpA*-NC was 0.04% smaller than Mu50 WT. However, the Mu50∆*lcpA*-LC colonies were not expanded when bacterial growth was inhibited by low temperature (4°C) from 24th to 48th hour (Figure 1D). These results suggest that the Mu50∆*lcpA*-LC had a growth advantage on solid medium at 37°C. However, the *lcpA* complementary strain Mu50∆*lcpA*-LC::*lcpA* still maintained the large colony morphology (Figure 1D), and the *lcpA* complementation did not restore the decreased pigment production of Mu50∆*lcpA*-LC but partially worked in Mu50∆*lcpA-*NC (Figure 1B). These results suggest that other unknown factors than the *lcpA* also contribute to the large colony morphology and decreased pigmentation of Mu50∆*lcpA*-LC.

In addition to the differences in colony morphology and pigmentation, Mu50∆*lcpA-*LC also showed a stronger spreading ability on soft agar plates in the spreading ability assay and decreased biofilm formation in the crystal violet staining assay when compared with Mu50∆*lcpA*-NC and Mu50 WT strains (Figures 1E,F). There were no differences in spreading ability and biofilm production between Mu50∆*lcpA*-NC and Mu50 WT (Figures 1E,F). Unexpectedly, the *lcpA* complementation resulted in a significantly lower biofilm production in Mu50∆*lcpA-*LC and Mu50∆*lcpA-*NC strains (Figure 1F). These results suggest that the stronger spreading ability and reduced biofilm formation helped Mu50∆*lcpA-*LC to expand on agar plates.

### Large and Normal Colony Isolates of Mu50∆*lcpA* Exhibit Distinct Susceptibility to β-Lactam Antibiotics

To test the resistance of Mu50∆*lcpA-*LC against β-lactam antibiotics, the MICs of all strains against three β-lactam, oxacillin, cefazolin, and penicillin were first determined by broth microdilution. As shown in Table 1, the BA01611∆*lcpA* had reduced MICs for all three β-lactam antibiotics and the complementation with *lcpA* gene in BA01611∆*lcpA* restored the decreased MICs. However, the Mu50 WT, Mu50∆*lcpA*-LC, Mu50∆*lcpA*-NC, and Mu50∆*lcpA*-NC::*lcpA* had same MICs for these β-lactams. Unexpectedly, Mu50∆*lcpA*-LC::*lcpA* displayed significantly decreased MICs of β-lactams (Table 1). This result revealed that the *lcpA* exerted strain-specific effects on β-lactam resistance in *S. aureus. lcpA* contributed to β-lactam resistance in BA01611, but it was not necessary for Mu50∆*lcpA*-NC. However, *lcpA* significantly decreased the β-lactam resistance in Mu50∆*lcpA*-LC.

**Table 1:** β-lactam MICs (μg/ml) of wild-type and *lcpA* mutant strains.

Strains	MIC (μg/ml)		
	Oxacillin	Cefazolin	Penicillin
BA01611	32	64	512
BA01611∆*lcpA*	16	16	256
BA01611∆*lcpA*∷*lcpA*	32	64	512
Mu50	256	128	16
Mu50∆*lcpA*	256	128	16
Mu50∆*lcpA-*LC	256	256	16
Mu50∆*lcpA-*NC	256	128	16
Mu50∆*lcpA*-LC∷*lcpA*	8	32	4
Mu50∆*lcpA*-NC∷*lcpA*	256	128	16

*LC, large colony; NC, normal colony*.

**Figure 2:**
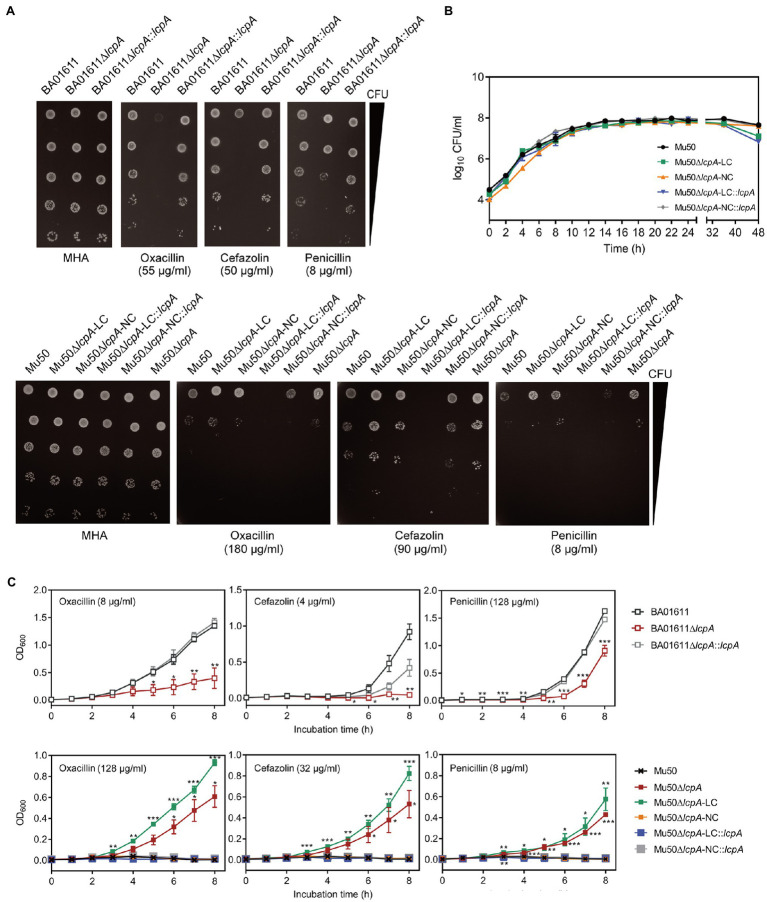
β-Lactam susceptibility assay for BA01611 and Mu50 isogenic strains. **(A)** β-Lactam resistance was measured by agar dilution for BA01611 and Mu50 isogenic strains. Bacteria cultures were diluted from 10^8^ to 10^4^CFU/ml, then 1μl of bacterial suspensions was spotted on the MHA+2% NaCl medium with different antibiotics. Images of MHA plates represent one out of three experiments showing similar results. **(B)** Growth curves of Mu50 isogenic strains in TSB without antibiotics showing by CFU/ml. **(C)** Growth curves of BA01611 and Mu50 isogenic strains in TSB with β-lactams showing by OD_600_. Error bars correspond to SEM from three independent experiments. One-way ANOVA with LSD or Dunnett’s T3 test for multiple comparisons, ^*^p<0.05, ^**^p<0.01, ^***^p<0.001.

The resistance of *lcpA* null mutants against antibiotics was further investigated by agar dilution, first using a multiple concentration gradient and then using a fixed concentration, at which the difference of resistance against the antibiotics was most obvious. As shown in Figure 2A, compared with the WT strains and complementary strains, the Mu50∆*lcpA*-LC had the highest resistances against β-lactams, while the resistances of Mu50∆*lcpA*-LC::*lcpA* decreased sharply. In contrast, the BA01611∆*lcpA* was more susceptible to β-lactams than its WT and complementary strains (Figure 2A). Besides, susceptibilities to glycopeptide antibiotics (vancomycin and teicoplanin), tetracycline, and clindamycin were also measured. The BA01611∆*lcpA* was more susceptible to vancomycin but not to teicoplanin than its WT and complementary strains. Mu50∆*lcpA*-LC was more sensitive to teicoplanin and vancomycin than its WT and complementary strains, while Mu50∆*lcpA*-NC was more resistant to vancomycin and more sensitive to teicoplanin (Supplementary Figure S1). β-Lactams and glycopeptides act on the cell wall, while the drug target site of tetracycline and clindamycin is the intracellular ribosome. Both BA01611∆*lcpA* and Mu50∆*lcpA*-NC showed increased susceptibility to tetracycline and clindamycin, while Mu50∆*lcpA*-LC had same resistances to these two antibiotics as the wild type (Supplementary Figure S1). These results showed again that the effect of deleting *lcpA* on antibiotic resistance was strain-specific. For BA01611, deleting *lcpA* reduced resistance to most cell wall-targeting antibiotics and ribosome-targeting antibiotics. For Mu50∆*lcpA*-NC, deleting *lcpA* had no significant effect on resistance to most cell wall-targeting antibiotics but decreased its ribosome-targeting antibiotic resistance. For Mu50∆*lcpA*-LC, deleting *lcpA* increased the resistance to all cell-wall targeted antibiotics but had no effect on resistance to ribosome-targeting antibiotics. These results suggested that Mu50∆*lcpA* could protect its bacterial population against not only cell-wall-targeted antibiotics but also ribosome-targeting antibiotics by switching into Mu50∆*lcpA*-LC.

To corroborate the result obtained by agar dilution, the growth curves in TSB containing β-lactam antibiotics were investigated. All Mu50 isogenic strains had same growth rates in TSB without antibiotics (Figure 2B). As shown in Figure 2C, the Mu50∆*lcpA*-LC strain had higher resistance than Mu50 WT evidenced by more growth advantages after 3h incubation, while the resistance to β-lactams of Mu50∆*lcpA*-NC was similar to Mu50 WT. The mixed strain Mu50∆*lcpA* (including Mu50∆*lcpA*-LC and Mu50∆*lcpA*-NC) also displayed higher antibiotic resistance than Mu50 WT and Mu50∆*lcpA*-NC but lower than Mu50∆*lcpA*-LC, suggesting the increased β-lactam resistance of Mu50∆*lcpA* was ascribed to the Mu50∆*lcpA*-LC cells. The growth of Mu50∆*lcpA*-LC::*lcpA* was inhibited in antibiotics (Figure 2C). These results prove again that the Mu50∆*lcpA*-LC had increased resistance against β-lactams. Collectively, deleting *lcpA* decreased β-lactam resistance in BA01611∆*lcpA* but increased β-lactam resistance in Mu50∆*lcpA-*LC, based on the agar dilution assay and the growth curve test.

### Most Mu50∆*lcpA*-LC Cells Possess a Complete Septum and Have a Large Cell Size

**Figure 3:**
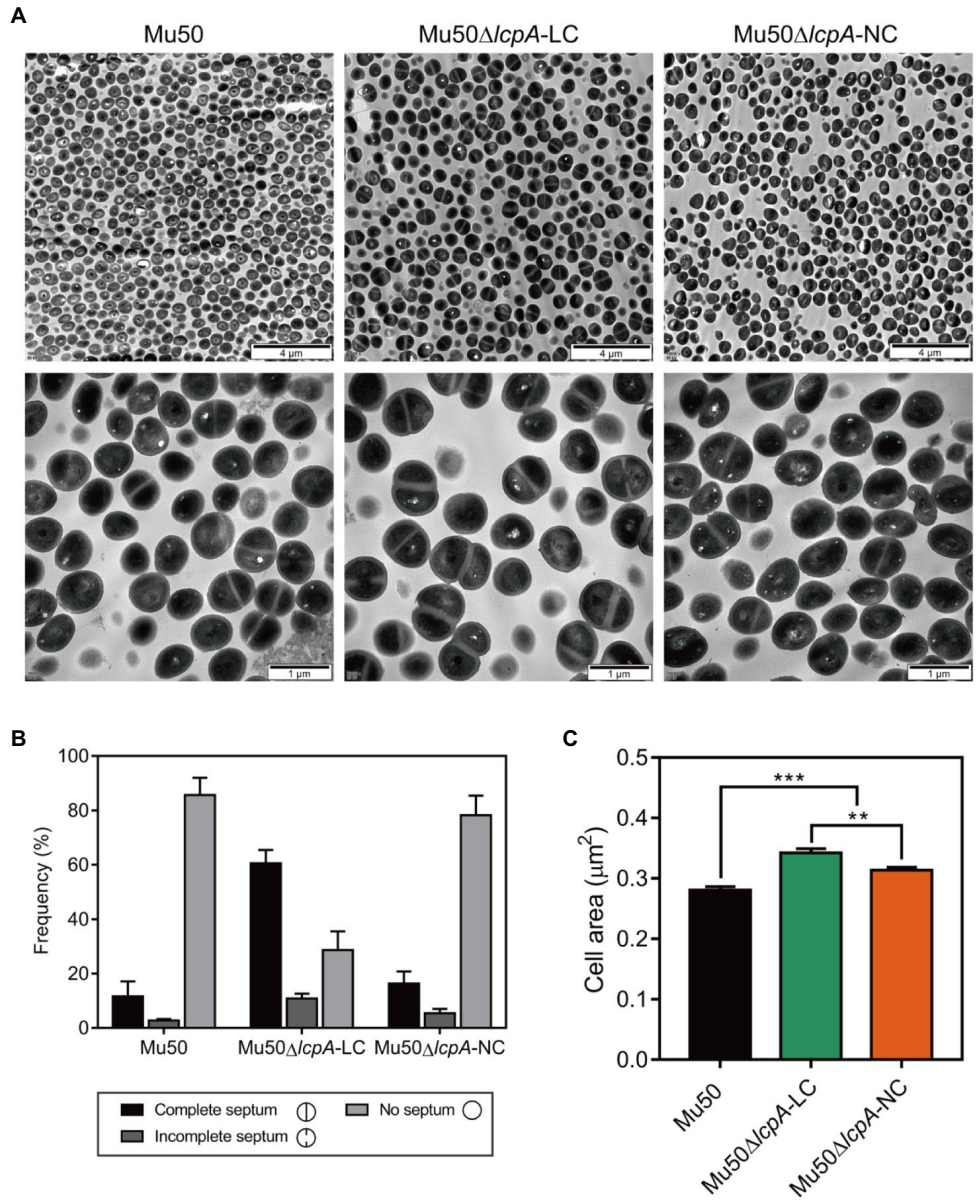
TEM reveals cell septal morphology of Mu50 isogenic strains. **(A)** TEM images of wild-type Mu50, Mu50∆*lcpA*-LC and Mu50∆*lcpA*-NC in TSB to stationary phase at 37°C. The scale bar of the top row of images is 4μm, and the scale bar of the bottom row of images is 1μm. **(B)** To estimate the fraction of the cells with the complete septum, at least 400 cells were scored according to the septum growth. **(C)** To estimate the individual cell size, the cell area of 300 cells per strain was measured. Error bars correspond to SEM. One-way ANOVA with LSD test for multiple comparisons, ^*^p<0.05, ^**^p<0.01, ^***^p<0.001.

To investigate the cell envelope changes in Mu50∆*lcpA-*LC, the cells of Mu50 WT, Mu50∆*lcpA-*LC, and Mu50∆*lcpA-*NC at the stationary phase were imaged by transmission electron microscopy (TEM). As shown in Figure 3A, no defect in cell walls in all strains was observed. However, a larger fraction of Mu50∆*lcpA-*LC cells had a complete closed septum compared with Mu50 WT and Mu50∆*lcpA-*NC. As quantified in Figure 3B, a complete septum was observed in 60.5±2.9% of the Mu50∆*lcpA-*LC cells compared to 16.3±2.7% of the Mu50∆*lcpA*-NC cells and 11.6±3.1% of the Mu50 WT cells. There were also many cells (10.8±1.7%) with the incomplete septum in Mu50∆*lcpA-*LC. In contrast, a large proportion of Mu50 WT cells (85.6±3.7%) and Mu50∆*lcpA*-NC cells (78.3±4.2%) were non-dividing, while only 28.7±4.0% of Mu50∆*lcpA-*LC cells had no septum. Furthermore, the individual cell sizes of Mu50∆*lcpA*-LC and Mu50∆lcpA-NC cells were 21.7 and 11.7% larger than that of the Mu50 WT (Figure 3C). In addition, Mu50∆*lcpA*-LC cell area was significantly larger than Mu50∆*lcpA*-NC (Figure 3C). These results suggest that most Mu50∆*lcpA-*LC cells with the closed septum may increase the cell size (Figure 3C) by halting in inward progression of cell splitting, which may contribute to the expansion of the large colonies.

### Mu50∆*lcpA*-LC Has a Significant High Level of *spa* Expression

**Figure 4:**
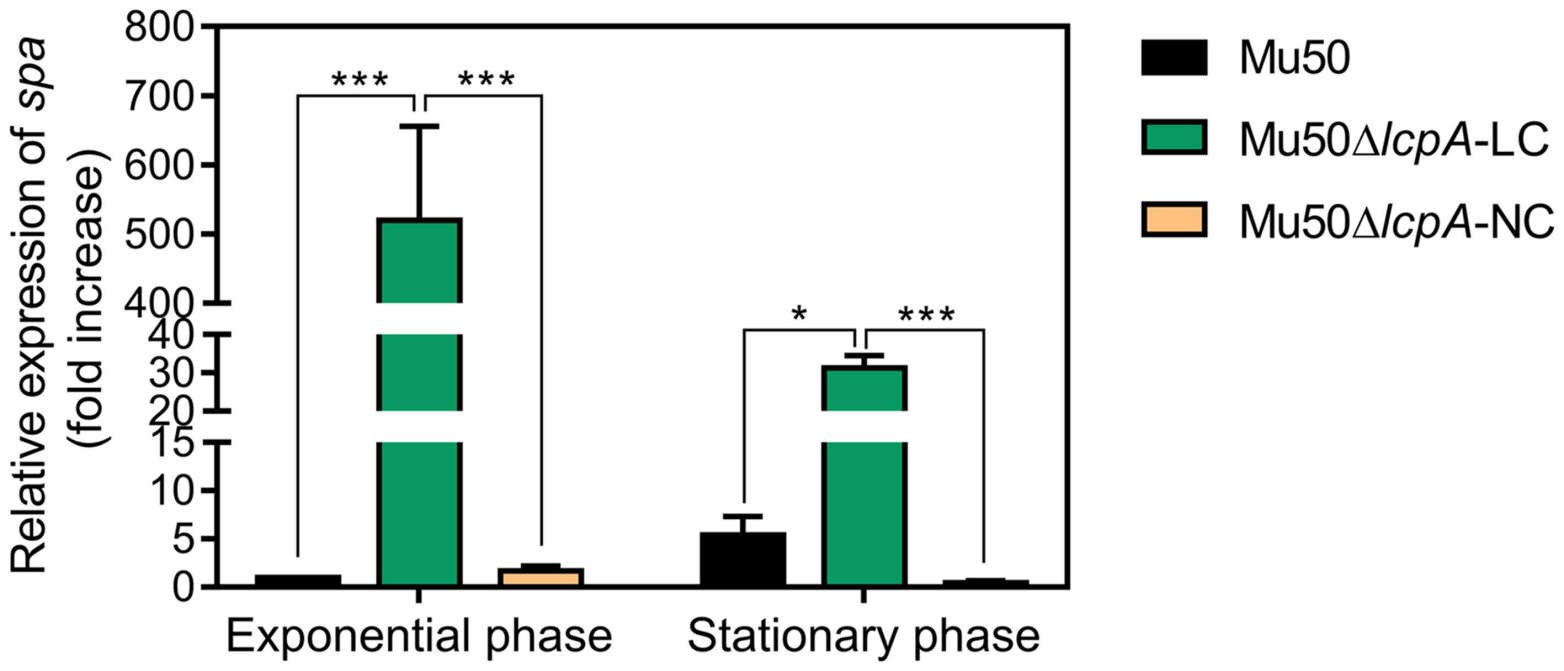
qRT-PCR analysis for expression of *spa* in Mu50 isogenic strains grown at the exponential phase and the stationary phase. Genes were normalized with the expression level of *gyrB*. Error bars correspond to SEM, five independent experiments. One-way ANOVA with LSD test for multiple comparisons, ^*^p<0.05, ^**^p<0.01, ^***^p<0.001.

Lower pigmentation, decreased biofilm formation, higher spreading ability and increased resistance to a Bsa bacteriocin that targets cell wall have been reported for a variant of a clinical MRSA strain, in comparison with its parent strain (Koch et al., 2014). The authors attribute the changes to a mutation in the *rsbW* gene, which upregulated the *agr* system, leading to changes in expression levels of *agr* downstream-regulated genes, such as a decrease in *spa* gene and an increase in *psm* gene (Koch et al., 2014). But Mu50 is an *agr*-defected strain although it possesses an *agr* system (Sakoulas et al., 2002). To investigate whether the phenotype switching of Mu50∆*lcpA-*LC was also induced by activating the *agr* system, we performed the qRT-PCR assay. The result showed that the *agr* system was not activated in Mu50∆*lcpA-*LC (data not shown). On the contrary, the expression of *spa* was significantly increased in Mu50∆*lcpA-*LC, in comparison with Mu50 WT, increased about five hundred times at the exponential phase and about 5.8 times at the stationary phase (Figure 4), respectively. These results suggest that the phenotype switching of Mu50∆*lcpA-*LC was not induced by upregulating *agr* system.

## Discussion

The phenotypic diversity can spontaneously emerge in an isogenic population, allowing bacteria to survive dynamic environments (Ackermann, 2015). Here, we first identified a reversible large colony variant Mu50∆*lcpA*-LC from an isogenic population of *S. aureus* Mu50∆*lcpA*. Mu50∆*lcpA*-LC is distinct broadly in phenotypes, with decreased susceptibility to β-lactam antibiotics, high spreading ability, reduced biofilm formation, low pigmentation, and a significant increase in *spa* expression level. Our results suggest that the *S. aureus* can evolve in response to antibiotic stress and host immunity system by phenotypic switching.

Mu50∆*lcpA*-LC forms large colonies due to its growth advantage on solid medium (Figure 1D). It has been known that the formation of SCVs has been directly associated with its slow growth (Proctor et al., 2006). In Mu50∆*lcpA*-LC, switching to the large colony phenotype may promote these bacterial cells to move to more suitable places for nutrition and colonization *in vivo*, due to their higher spreading ability and decreased biofilm formation (Figures 1E,F). The high spreading ability promoted motility of bacterial cells on surfaces (Koch et al., 2014). The less polymeric matrix of biofilm reduced restrictions on the expansion of colony on 1% agar plate in *Vibrio cholerae* (Yan et al., 2017). In addition, the TEM assay showed that the individual cell size of Mu50∆*lcpA*-LC was 21.7% larger than that of Mu50 WT, while the colony diameter of Mu50∆*lcpA*-LC was 66.4% larger than that of Mu50 WT. Importantly, Mu50∆*lcpA*-LC did not differ from WT in terms of the growth rate (Figure 2B). It has been reported that a phase variant with an unchanged growth rate could expand rapidly and form large colonies due to its elongated cells and cell chains in rod bacteria *Clostridioides difficile* (Garrett et al., 2019). On the other hand, Kriegeskorte et al. (2014) reported that the enlarged cells with incomplete or multiple division planes of ∆*thyA* mutant of *S. aureus* could form small colonies, possibly due to the lower growth rate and severe growth defects of the mutant. Therefore, the large cell size of Mu50∆*lcpA*-LC, combined with its high spreading ability and low biofilm formation, could contribute to the colony expansion.

Mu50∆*lcpA*-NC had same MICs against all three β-lactam antibiotics, while BA01611∆*lcpA* reduced MICs against all three β-lactam antibiotics (Table 1). Decreased resistance of BA01611∆*lcpA* could be due to the decreased level of PGN cross-linking that was controlled by WTA (Atilano et al., 2010). However, Mu50 could resist *lcpA* deletion through its active PGN synthesis and thicker cell wall, as observed by Cui et al. (2000). The TEM assay also proved that lacking *lcpA* did not lead to a defective cell wall in Mu50∆*lcpA*-NC (Figure 3A). Broth microdilution showed that Mu50∆*lcpA*-LC had same β-lactam MICs as WT and Mu50∆*lcpA*-NC strains (Table 1). The precision of broth microdilution was considered to be plus or minus 1 twofold concentration. To increase the precision, we used agar dilution and the growth curve method, with more concentration gradients. The increased antibiotic resistance of Mu50∆*lcpA*-LC against β-lactams was demonstrated by agar dilution and the growth curve test (Figure 2). The high resistance could result from by phenotype switching. The TEM confirmed that most Mu50∆*lcpA-*LC cells possessed complete septum even at the stationary phase (Figure 3), which could reduce the access of antibiotics to its lethal target at the division septum, where cell walls are synthesized.

Several phenotypic changes we observed in Mu50∆*lcpA-*LC are similar to what has been reported for an un-pigmented variant “W” developed from a clinical MRSA strain (Koch et al., 2014). Both Mu50∆*lcpA*-LC and the “W” variant had a high spreading ability, decreased biofilm formation and low pigmentation. However, mechanisms for such phenotypic changes could be different. The “W” variant produced the lantibiotic bacteriocin of *S. aureus* (Bsa) which targets bacterial cell wall precursors to remove niche competitors in the intra-clonal competitive environment (Koch et al., 2014). However, Mu50 does not contain the *bsa* gene cluster and had a growth advantage in cell wall-targeting antibiotics (Figure 2). In addition, the “W” variant with *RsbW* mutation caused hyperactivation of the *agr* system (Koch et al., 2014). However, Mu50 is an *agr*-deficient strain that carries several silent mutations in *agrA* (Sakoulas et al., 2002). Indeed, in this study, all the Mu50 isogenic strains express the *RNAIII* at extremely low levels (data not shown). Furthermore, the *spa* gene expression, which was inhibited by *agr* in the “W” variant, was unexpectedly increased significantly in Mu50∆*lcpA-*LC than other isogenic strains at the exponential and stationary phases (Figure 4). Despite unknown mechanisms for phenotypic changes in Mu50∆*lcpA-*LC, the activation of *spa* expression may allow Mu50∆*lcpA-*LC to escape the host immunity, since *spa* encodes the surface protein A that binds the Fc region of immunoglobulin G (IgG) and facilitates the bacteria evasion from the host immune response (Atkins et al., 2008).

The changed phenotypes of Mu50∆*lcpA-*LC, including the possession of septum at the stationary phase, β-lactam antibiotic resistance, spreading ability, the staphyloxanthin production (a component of cell functional membrane microdomain) and the upregulated *spa* encoding the surface protein A are all associated with the cell wall structure (Kaito and Sekimizu, 2007; García-Fernández et al., 2017). Thus, it is plausible that the distinct phenotypes of Mu50∆*lcpA-*LC are associated with alteration of its cell envelope. Besides, phenotypic diversity could also occur independently of genetic variation by methods such as epigenetic modification of chromatin and stochastic gene expression (Ackermann, 2015). Therefore, further investigation is needed to find the mechanisms of the phenotypic diversity of Mu50∆*lcpA*.

**Figure 5:**
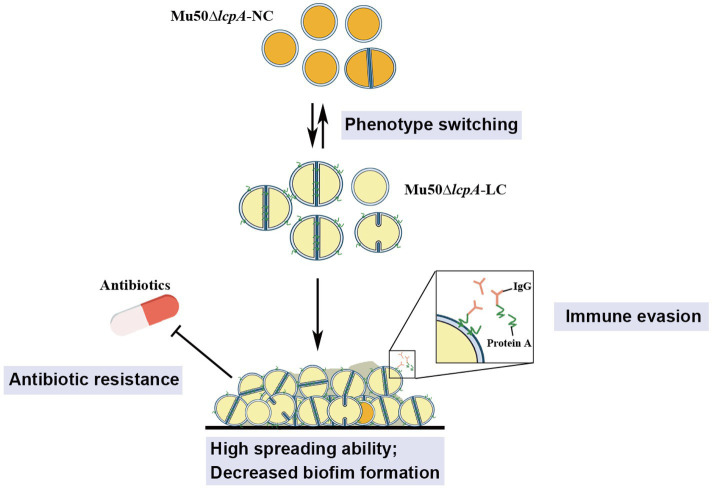
Illustration of the phenotype heterogeneity in an isogenic population of *S. aureus*. The population of MRSA Mu50∆*lcpA* consists of two subpopulations that produce large colony morphology (Mu50∆*lcpA*-LC) and normal colony morphology (Mu50∆*lcpA*-NC) on agar plates. These two morphologies occur reversibly. Most Mu50∆*lcpA*-LC cells possess a complete septum and have a large cell size. Importantly, this less pigmented Mu50∆*lcpA*-LC exhibited a high spreading ability on solid media and had increased resistance to β-lactam antibiotics. Moreover, upregulated *spa* expression suggests enhanced ability of Mu50∆*lcpA*-LC to evade immune responses. This phenotypic switching will help *S. aureus* to survive complex host environments.

These findings from this study will aid in our understanding of the ability of *S. aureus* to adapt to fluctuating environments through the development of strain-level variation. The variant in this study may allow *S. aureus* community to evade environmental stresses such as antibiotics and immune system to colonize rapidly in hosts, causing persistent infections (Figure 5).

## Data Availability Statement

The original contributions presented in the study are included in the article/Supplementary Material, further inquiries can be directed to the corresponding author.

## Author Contributions

XZ and YS conceived and designed the experiments and wrote the manuscript. YS collected the data and performed the analysis. ML and MN participated in data collection. All authors have read and approved the final manuscript.

## Funding

This work was supported by the National Key Research and Development Program of China (2016YFD0500507) and Shaanxi Key Research and Development Program (2020NY - 189).

## Conflict of Interest

The authors declare that the research was conducted in the absence of any commercial or financial relationships that could be construed as a potential conflict of interest.

## Publisher’s Note

All claims expressed in this article are solely those of the authors and do not necessarily represent those of their affiliated organizations, or those of the publisher, the editors and the reviewers. Any product that may be evaluated in this article, or claim that may be made by its manufacturer, is not guaranteed or endorsed by the publisher.
