# Supplementary material for: Phenotypic Switching of Staphylococcus aureus Mu50 Into a Large Colony Variant Enhances Heritable Resistance Against β-Lactam Antibiotics
Source: Front Microbiol. 2021 Oct 7;12:709841. doi: 10.3389/fmicb.2021.709841 (PMC8530407; doi:10.3389/fmicb.2021.709841)
Supplement: Supplementary file 1 [file Data_Sheet_1.ZIP › Supplemental material presentation/Figure S1.pdf]

Supplemental material

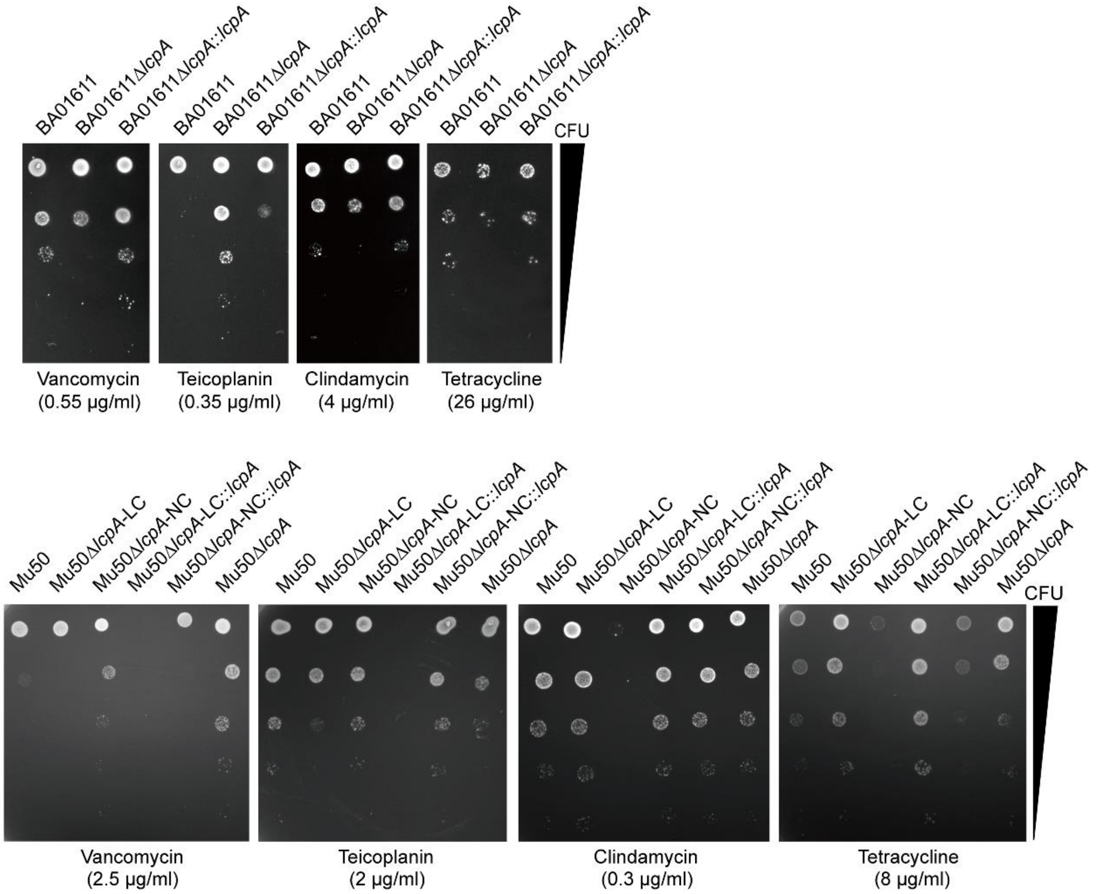

FIG S1 Antibiotic susceptibility assay testing by the agar dilution method for BA01611 and Mu50 isogenic strains. Bacteria cultures were diluted from 10^8 to 10^4 CFU/ml, then one microliter of bacterial suspensions was spotted on the MHA + 2% NaCl medium with different antibiotics. Images of MHA plates represent one out of three experiments showing similar results.
