# Supplementary material for: Phenotypic Switching of Staphylococcus aureus Mu50 Into a Large Colony Variant Enhances Heritable Resistance Against β-Lactam Antibiotics
Source: Front Microbiol. 2021 Oct 7;12:709841. doi: 10.3389/fmicb.2021.709841 (PMC8530407; doi:10.3389/fmicb.2021.709841)
Supplement: Supplementary file 1 [file Data_Sheet_1.ZIP › Supplemental material presentation/TABLE S1.pdf]

Supplemental material

TABLE S1 Strains and plasmids used in this study

Strains and plasmids	Description	Source
***Escherichia coli* strains**		
DH5α	F-ϕ80(*lac*Z)ΔM15 Δ(*lac*ZYA-*arg*F)U169 *end*A1 *rec*A1 *hsd*R17(r_K^-, m_K^+) *sup*E44λ-*thi*-1 *gyr*A96 *rel*A1 *pho*A	Laboratory strain
IM08B	DC10B, ΩP_{help}-*hsdMS* (CC8-2), ΩP_{N25}-*hsdMS* (CC8-1), modifies plasmids to carry staphylococcal specific methylations	Laboratory strain
***Staphylococcus aureus* strains**		
Mu50	MRSA clinical strain, carrying *lcpA* (E146K), *mecA*$^+$	Laboratory strain
Mu50Δ*lcpA*	Mu50 *lcpA* null mutant having LC and NC forms	This study
Mu50Δ*lcpA*-LC	A derivative of Mu50Δ*lcpA* with large colony morphology	This study
Mu50Δ*lcpA*-NC	A derivative of Mu50Δ*lcpA* with normal colony morphology	This study
Mu50Δ*lcpA*-LC::*lcpA*	Complementation strain	This study
Mu50Δ*lcpA*-NC::*lcpA*	Complementation strain	This study
BA01611	A bovine-associated MRSA strain, *mecA*$^+$	Laboratory

		strain
BA01611Δ*lcpA*	BA01611 *lcpA* null mutant	This study
BA01611Δ*lcpA*::*lcpA*	Complementation in BA01611 *lcpA* null mutant	This study
**Plasmids**		
pKZ2	*E. coli* - *S. aureus* shuttle vector with a thermosensitive origin of replication for construction of the allelic replacement	Laboratory plasmid
pΔ*lcpA*	Knockout plasmid	This study
pΔ*lcpA*-*lcpA*^{E146K}	Complementary plasmid for Mu50Δ*lcpA*	This study
pΔ*lcpA*-*lcpA*	Complementary plasmid for BA01611Δ*lcpA*	This study
