# Supplementary material for: Phenotypic Switching of Staphylococcus aureus Mu50 Into a Large Colony Variant Enhances Heritable Resistance Against β-Lactam Antibiotics
Source: Front Microbiol. 2021 Oct 7;12:709841. doi: 10.3389/fmicb.2021.709841 (PMC8530407; doi:10.3389/fmicb.2021.709841)
Supplement: Supplementary file 1 [file Data_Sheet_1.ZIP › Supplemental material presentation/TABLE S2.pdf]

Supplemental material

TABLE S2 Primers used in this study

Primer	Sequence (5’-3’)*
P1	AGCCTCGGAACCGGTACCGGATTCGCTTTGGGAATCACTTTCTAAAGTAATG
P2	GAAATCGCCTTGTTTATTTAGCAAGTGATTTCTTAATAAATCGTAC
P3	AAGAAATCACTTGCTAAATAAACAAGGCGATTTCTATCATACTC
P4	TCTCCGGCGGCCGCTCGGAAGCCCACAGTACAATAAATGCC
P5	GAGAACGACTTGCAGGCCGTGTC
P6	CTCGCAAGGGCTGAATTGGCC
P7	TGACGTTGAGCCTCGGAACCGGTACCGGTCTATATCGTTTCCAAGGATTAC
P8	GAAATCGCCTTGTTTATTTCTCGAGTTAATCTTCATCTAAAAAGTCTTTAATAGCT
P9	TTGACGTTGAGCCTCGGAACCGGTACCGGTCCAAGTGCTAAGAGGTATACAGTTATGC
P10	CTTTTTAGATGAAGATTAACTCGAGAAATAAACAAGGCGATTTCTATCATACTC
P11	GATCTCCGGCGGCCGCTCGGAATTCTGCCCACAGTACAATAAATGCCTG
RT-*gyrB*-F	TTATGGTGCTGGGCAAATACAAG
RT-*gyrB*-R	CCACACTAAATGGTGCAAACCTC

RT-*spa*-F	GAATCTCAAGCACCGAAAGCGG
RT-*spa*-R	CTTTGCTCACTGAAGGATCGTC

*Restriction sites (*Xho*1) are underlined.
